# Co‐culture of pro‐inflammatory macrophages and myofibroblasts: Evaluating morphological phenotypes and screening the effects of signaling pathway inhibitors

**DOI:** 10.14814/phy2.14704

**Published:** 2021-01-19

**Authors:** Colin Venter, Kathryn H. Myburgh, Carola U. Niesler

**Affiliations:** ^1^ Discipline of Biochemistry School of Life Sciences University of KwaZulu‐Natal Scottsville South Africa; ^2^ Department Physiological Sciences Stellenbosch University Matieland South Africa

**Keywords:** cell‐cell communication, cellular phenotype, intercellular communication, PI3kinase inhibitor, skeletal muscle myoblasts, smooth muscle actin

## Abstract

Skeletal muscle regeneration is a complex process influenced by non‐myogenic macrophages and fibroblasts, which acquire different phenotypes in response to changes in the injury milieu or changes in experimental conditions. In vitro, serum stimulates the differentiation of fibroblasts into myofibroblasts, while lipopolysaccharide (LPS) stimulates the polarization of unstimulated (M0) macrophages to acquire an M1 pro‐inflammatory phenotype. We characterized these phenotypes using morphology (with circularity as shape descriptor; perfect circularity = 1.0) and phenotype‐specific markers. Myofibroblasts (high α‐smooth muscle actin [SMA] expression) had high circularity (mean 0.60 ± 0.03). Their de‐differentiation to fibroblasts (low α‐SMA expression) significantly lessened circularity (0.47 ± 0.01 and 0.35 ± 0.02 in 2% or 0% serum culture media respectively (*p* < 0.05). Unstimulated (M0) macrophages (no CD86 expression) had high circularity (0.72 ± 0.02) which decreased when stimulated to M1 macrophages (CD86 expression) (LPS; 0.61 ± 0.02; *p* < 0.05). Utilizing these established conditions, we then co‐cultured M1 macrophages with myofibroblasts or myoblasts. M1 macrophages significantly decreased relative myofibroblast numbers (from 223 ± 22% to 64 ± 7%), but not myoblast numbers. This pro‐inflammatory co‐culture model was used to rapidly screen the following four compounds for ability to prevent M1 macrophage‐mediated decrease in myofibroblast numbers: L‐NAME (inducible nitric oxide synthase inhibitor), SB203580 (p38 mitogen‐activated protein kinase inhibitor), SP600125 (c‐Jun N‐terminal kinase inhibitor) and LY294002 (phosphoinositide 3‐kinase [PI3K] inhibitor). We found that LY294002 rescued myofibroblasts and decreased macrophage numbers. Myofibroblast rescue did not occur with L‐NAME, SB203580 or SP600125 incubation. In conclusion, these data suggest a PI3K‐associated mechanism whereby myofibroblasts can be rescued, despite simulated pro‐inflammatory conditions.

## Graphical abstract

In the current study we established the exact conditions for generating, and tools for morphologically evaluating and characterizing, specific macrophage and fibroblast phenotypes. Subsequently, we used these conditions to investigate the effect of M1 macrophages on myofibroblast and myoblast numbers, and then screen for inhibitors that could abrogate the observed negative effect of the pro‐inflammatory macrophages on myofibroblasts.

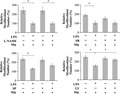

## 1 INTRODUCTION

Skeletal muscle repair involves the proliferation, differentiation, and fusion of muscle stem cells (myoblasts) into regenerated muscle fibers. This process, known as myogenesis, is regulated in part by cell‐cell communication between myoblasts and non‐myogenic cells (including macrophages and fibroblasts), as well as communication between the non‐myogenic cells themselves (Bentzinger et al., 2013). During in vivo myogenesis, relatively quiescent fibroblasts differentiate into activated myofibroblasts; this is influenced by macrophages (Bosurgi et al., (2011); Mann et al., 2011). Unstimulated (M0) macrophages are present during the early stages of wound repair and acquire a pro‐inflammatory (M1) phenotype to produce factors such as tumor necrosis factor (TNF)‐α and nitric oxide (NO). These cells later switch to an anti‐inflammatory (M2) phenotype to secrete pro‐fibrotic factors (such as transforming growth factor [TGF]‐β) to activate fibroblasts to differentiate into an intermediate proto‐myofibroblast phenotype (absent of contractile apparatus) before fully and reversibly differentiating into contractile myofibroblasts (Arnold et al., 2007; Desmouliere et al., 1993; Tomasek et al., 2002). Myofibroblasts are primarily responsible for depositing matrix factors to re‐establish the extracellular matrix surrounding healing muscle fibers, but also regulate muscle regeneration (Chapman et al., 2016; Rao et al., 2013).

Fibroblasts and macrophages can also acquire different phenotypes depending on the in vitro experimental conditions (Desai et al., 2014; Ploeger et al., 2013). Fibroblasts become activated by serum to reversibly differentiate to myofibroblasts (Hecker et al., 2011; Howard et al., 2012). M0 macrophages typically respond to an inflammatory stimulus, such as lipopolysaccharide (LPS), to acquire an M1 phenotype (Bosurgi et al., 2011). Studies often distinguish between the macrophage phenotypes (Ploeger et al., 2013; Tarique et al., 2015; Villalta et al., 2009), but have only more recently begun to make the distinction between fibroblasts and myofibroblasts (Baum & Duffy, 2011; Bochaton‐Piallat et al., 2016). Furthermore, cells often acquire different morphologies associated with their phenotype and thus rapid assessment and quantification of cell shapes may be a useful tool to evaluate the effect of experimental conditions on the cells. Therefore, we first sought to establish different phenotypes of macrophages and fibroblasts and characterize their morphologies.

Intercellular communication between the different macrophage and fibroblast phenotypes has knock‐on effects regulating myogenesis; a dysregulated communication leads to aberrant muscle wound repair (Bosurgi et al., 2011; Mann et al., 2011). A chronic presence of M2 macrophages leads to a pro‐fibrotic microenvironment, and this appears to be mediated by soluble factors (such as TGF‐β) and/or activation of signaling pathways (such as the Smad pathway) in the target fibroblasts (Biernacka et al., 2011). It is still unclear what the effect of a chronic presence of M1 macrophages is on fibroblasts and by implication on myogenesis. We have previously shown that myofibroblasts promoted the fusion of myoblasts (Venter & Niesler, 2018a); pro‐inflammatory macrophage‐secreted soluble factors have been shown to induce fibroblast death (Huang et al., 2009; Humphreys & Wilson, 1999; Nascimento et al., 2015). However, direct contact has also been shown to regulate interaction between macrophages and fibroblasts (Steinhauser et al., 1998). We therefore used our previously developed co‐culture method, which keeps the cell types separate but permits some degree of cell contact, to co‐culture M1 macrophages with myofibroblasts or myoblasts. This was then used as a model to rapidly screen for inhibitors that could prevent the negative effects of M1 macrophages on myofibroblasts. The inhibitors L‐NAME, SB203580, SP600125, and LY294002 targeted inducible nitric oxide synthase (iNOS), mitogen‐activated protein kinase (MAPK), c‐Jun N‐terminal kinase (JNK), and phosphoinositide 3‐kinase (PI3K), respectively. Our approach offered a rapid screen for signaling pathway inhibitors that mediate macrophage‐dependent regulation of myofibroblast number in vitro.

## 2 METHODS

### 2.1 Reagents

Lipopolysaccharide (1 mg/ml; Capital Lab Supplies, cat. L4391) and L‐NAME (200 mM; BioVision, cat. 2356) were prepared in distilled water. SB203580 (10 mM; Santa Cruz, cat. SC‐3533), LY294002 (10 mM; Santa Cruz, cat. SC‐201426), and SP600125 (100 mM; Santa Cruz, cat. SC‐200635) were prepared in DMSO (Sigma, cat. D2650). Fuchsine (1% w/v; Capital Lab Supplies, cat. 47860) was dissolved in 100% methanol. Serum‐free medium (SFM) was prepared by supplementing Dulbecco's Modified Eagle's Medium (DMEM, Capital Lab Supplies, cat. D5648) with 2% (v/v) Penicillin‐Streptomycin (PenStrep, LONZA, cat. DE17‐602E). Serum‐containing medium (SCM) was prepared by supplementing SFM with 2% (v/v) or 10% (v/v) fetal bovine serum (Gibco, cat. 10500).

### 2.2 Mono‐cultures to establish conditions

Mouse C2C12 myoblasts (ATCC, cat. CRL‐1772™; passage 10–20), LM(TK) [LM(tk), LMTK] myofibroblasts (ATCC, USA, cat. CCL‐1.3™; passage 6–25), and J774A.1 macrophages (ATCC, cat. TIB‐67™; passage 60–90) were cultured at 37°C and 5% CO_2_ and maintained in 10% SCM which was changed every 48 h. Macrophages (40 × 10^3^) or myofibroblasts (30 × 10^3^) were seeded into 24‐well culture plates in 10% SCM (500 µl) and incubated for 2 h to promote adherence. The cells were then washed twice with sterile PBS (500 µl) and thereafter the myofibroblasts were treated with different concentrations of SCM (0, 2 and 10%; 500 µl) and macrophages with different concentrations of LPS (0 and 0.1 µg/ml; 500 µl) in 2% SCM for 24 h. Early myofibroblasts (so‐called proto‐myofibroblasts) were present in 2% SCM and fully differentiation myofibroblasts were identified in 10% SCM media, while fibroblasts were present in 0% SCM (i.e. SFM); M0 macrophages were stimulated to M1 macrophages in the presence of LPS.

### 2.3 Co‐cultures

Co‐cultures of two cell types were performed as previously described (Venter & Niesler, 2018b). To evaluate the effect of M1 macrophages on myofibroblasts or myoblasts, M0 macrophages (0 or 40 × 10^3^) were first plated on the outer edge of a 24‐well plate in 10% SCM and incubated for 1 h. Myofibroblasts (30 × 10^3^) or myoblasts (5 × 10^3^) were then plated in the center of the well and incubated for 2 h. The cells were then washed twice with sterile PBS (500 µl) and cultured in 2% SCM without LPS or with LPS treatment (0.1 µg/ml) for 24 h. To evaluate the mechanism whereby M1 macrophages may mediate changes in the myofibroblast proliferation, macrophages (40 × 10^3^) were co‐cultured with myofibroblasts (30 × 10^3^) using the following sequential protocol for manipulation: plating of the two cell types as described above; treatment with an inhibitor (L‐NAME, SB203580, SP600125, and LY294002) in 2% SCM for 30 min; thereafter, treatment with (1 h) LPS with or without inhibitor. The cells were then washed twice with sterile PBS and incubated further for 24 h with inhibitor, but without LPS. To evaluate the effect of LY294002 on macrophages, M0 macrophages (40 × 10^3^) mono‐cultures were treated with LY294002 for 24 h. To assess DMSO cytotoxicity, M0 macrophages and myofibroblasts co‐cultured with M1 macrophages were cultured with different dilutions of DMSO (0, 1:1000 and 1:10,000) in 2% SCM for 24 h.

### 2.4 Morphology analysis

Mono‐cultures and/or co‐cultures of cells were briefly washed with PBS and stained with 1% Fuchsine (10 min), submerged in water to remove excess the stain and left to dry. Cells were visualized and captured with an Olympus CKX41 microscope and a Motic 3.0‐megapixel camera (10× objective lens; five randomly selected fields of view per replicate for two replicates per experiment). Morphology was quantitively analyzed by assessing cell circularity (circularity = 4*π* × ([Area]/[Perimeter]^2^) in ImageJ. ImageJ was first set to include circularity analysis (*Analyze→Set Measurements*; check *Shape descriptors*). Images were first converted to 8‐bit (*Image→Type→8*‐*bit*), converted to a binary image (*Process→Binary→Make Binary*) and analysed (*Analyze→Analyze Particles*). The size was set between 100 and 800 pixels to limit background and excluded touching cells that prevented accurate analysis. The result was a value between 0 and 1 which indicated cells that are irregularly shaped or perfectly circular, respectively.

### 2.5 Proliferation analysis

Proliferation studies were analyzed as previously described (Venter & Niesler, 2019). Images of stained cells (4× objective lens; five randomly selected fields of view per replicate for two replicates per experiment) were automatically identified using ImageJ by converting the captured image to grayscale (*Image→Type→8*‐*bit*), removing the image noise (*Process→Noise→Despeckle*), adjusting the brightness and contrast (*Image→Adjust→Brightness*/*Contrast*: *min = 87*; *max = 167*), and finally applying first a Phansalkar threshold (*Image→Adjust→Auto Local Threshold*: *Phansalkar*), and then a watershed (*Process→Binary→Watershed*). The identified cells were then automatically quantified (*Analyze→Analyze Particles*).

### 2.6 Confocal microscopy

For mono‐culture phenotype assessment, myofibroblasts (30 × 10^3^) were plated in 24‐well plates on glass coverslips in 10% SCM for 3 h, washed twice with PBS and switched to 0%, 2% and 10% SCM for 24 and 72 h. Fresh media was added at 48 h. Macrophages (40 × 10^3^) were similarly plated in 2% SCM with or without 0.1 µg/ml LPS for 24 h. The media was then removed, the cells fixed with 4% paraformaldehyde (10 min), permeabilized with 0.3% Triton‐X100 (Sigma, cat. X100; 10 min), and blocked with 5% donkey serum (Sigma, cat. D9663) for 30 min at room temperature. The cells were then incubated overnight at 4°C with mouse anti‐α‐smooth muscle actin (SMA; 1:1000; Sigma, cat. A2547), washed with PBS (3 × 5 min) and incubated at room temperature (1 h) in the dark with Dylight594‐conjugated donkey anti‐mouse antibody (1:1000; Jackson ImmunoResearch, cat. 715‐515‐151). Macrophages were incubated with PE‐conjugated rat anti‐CD86 (1:200; BioLegend, cat. 105008) overnight at 4°C. Hoechst (1:100; 10 mg/ml; Sigma, cat. B2261) was subsequently added for 10 min and the coverslips washed with PBS (6 × 5 min) and mounted on glass slides with Mowiol (Sigma, cat. 81381). The cells were viewed with a Zeiss 710 confocal microscope (Carl Zeiss GmbH).

### 2.7 Statistical analysis

Data were determined to be normally distributed; all results were analyzed using a one‐way ANOVA with post hoc tests in GraphPad Prism 8 and values of *p* < 0.05 were considered to be statistically significant compared to the control. All data were represented as mean ± SEM.

## 3 RESULTS

### 3.1 Phenotypic characterization of macrophage and fibroblast populations with quantifiable characteristic of circularity

**FIGURE 1:**
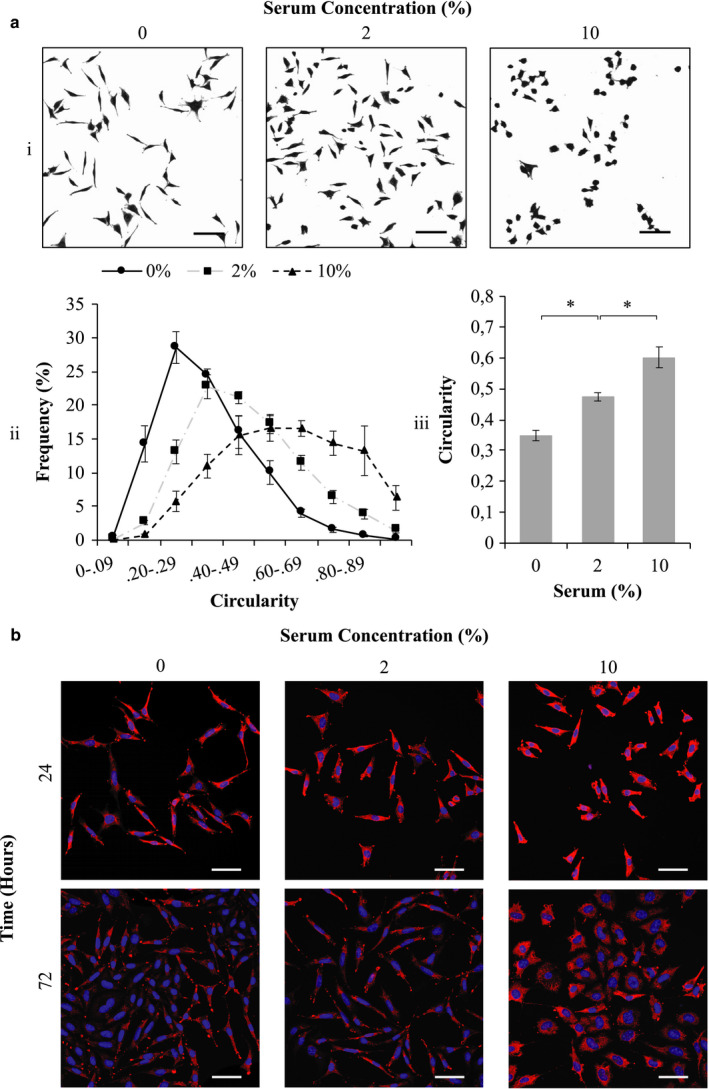
Characterization of fibroblast population phenotype in different concentrations of serum‐containing medium (SCM). (a) Morphological analysis of LMTK cells maintained in 0%, 2%, and 10% SCM showing (i) Fuchsine‐stained cells, (ii) circularity frequency distribution of these cells, as well as the average circularity of (iii) LMTK. (b) Confocal microscopy of fibroblast populations immunostained with mouse anti‐α‐SMA (red) maintained in different concentrations of SCM for 24 and 72 h. Hoechst was used as a nuclear stain (blue). Images were captured using an Olympus CKX41 microscope coupled to a Motic 3.0‐megapixel camera (4× objective lens; scale bar = 200 µm) and a Zeiss 710 confocal microscope (25× objective lens; scale bar = 50 µm). *p < 0.05; *N* = 4–7

Fibroblasts have been shown to reversibly differentiate into myofibroblasts in the presence of serum and/or TGF‐β in vitro (Hecker et al., 2011; Howard et al., 2012; Vaughan et al., 2000). LMTK cells were cultured in 0%, 2%, and 10% SCM for 24 h and phenotypes characterized based on morphology and α‐SMA (Figure 1). Cells cultured in media containing 0% serum acquired an elongated, thin morphology compared to those maintained in 10% SCM, which were clustered with thick, rounded shapes (Figure 1Ai); cells switched to 2% SCM had an intermediate morphology. Circularity was assessed to quantify these changes in morphology (Figure 1Aii). Cells maintained in 10% SCM had a peak frequency of circularity at 0.60–0.69. In response to 2% SCM, the peak frequency shifted to a circularity of 0.30–0.39, while 0% serum shifted the population further where the peak frequency of circularity was observed at 0.20–0.29. The average cell circularity for each serum concentration was subsequently determined. Under control conditions in 10% SCM, it was 0.60 ± 0.03, which decrease significantly in response to 2% SCM (*p* < 0.05) and progressively a further significant decline in 0% SCM (*p* < 0.05 compared to 2% SCM; Figure 1Aiii). Confocal microscopy was used to evaluate changes in α‐SMA expression of the myofibroblast population following incubation in the absence or presence of serum (0%, 2%, and 10%; Figure 1B). Although myofibroblasts cultured in media containing either 0% or 2% serum still expressed α‐SMA, the level of expression appeared lower at 72 h, when compared to those cells cultured in the presence of 10% serum. In addition, cells cultured under low serum conditions morphologically displayed longer, thinner shapes, whereas those culture in the presence of 10% serum were much rounder. We therefore concluded that following culture in the absence of serum, myofibroblasts begin to decrease their α‐SMA expression and display a lower circularity, indicative of a transition to a fibroblast phenotype; in the presence of 10% serum, the expression of α‐SMA and a higher circularity support their identification as myofibroblasts. Those exposed to 2% serum, although showing a decreased circularity and α‐SMA expression, were rounder than cells cultured in the absence of serum, suggesting partial de‐differentiation to proto‐myofibroblasts, an intermediate phenotype characteristic of early myofibroblasts (Hinz et al., 2007).

**FIGURE 2:**
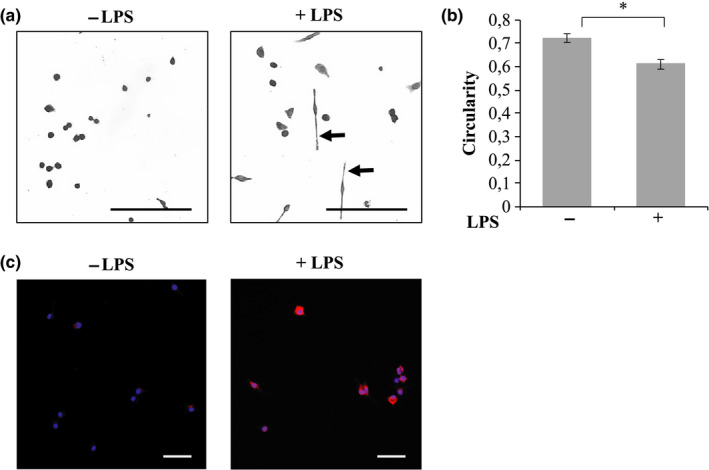
Characterization of macrophages and myofibroblasts in the presence of lipopolysaccharide (LPS). Morphological analysis of cells maintained in 2% serum‐containing medium with and without LPS (0.1 µg/ml) showing (a) Fuchsine‐stained macrophages and the average circularity of (b) macrophages. (c) Confocal microscopy of macrophages immunostained with rat anti‐CD86 (red) treated with LPS for 24 h. Hoeschst was used as a nuclear stain (blue). Images were captured using an Olympus CKX41 microscope coupled to a Motic 3.0‐megapixel camera (10× objective lens; scale bar = 200 µm) and a Zeiss 710 confocal microscope (25× objective lens; scale bar = 50 µm). *p < 0.05; *N* = 6

To establish a population of M1 macrophages, J774a.1 cells were cultured in the presence of 2% SCM and LPS for 24 h; phenotype was then characterized based on morphology and CD86 expression (Figure 2). Macrophages cultured in the absence of LPS were small and rounded, while the cells cultured in the presence of LPS had acquired long bipolar protrusions (Figure 2a). The average circularity of macrophages was then calculated (Figure 2b) as follows: macrophage circularity in the absence of LPS was 0.72 ± 0.02; this decreased significantly to 0.61 ± 0.02 when treated with LPS (Figure 2b; (*p* < 0.05). Macrophages stimulated with LPS also displayed a notable increase in CD86 expression compared to unstimulated cells (Figure 2c). The increased CD86 staining density was mostly restricted to central regions and not the protrusions. These results suggest that stimulation with LPS was able to polarize the J774a.1 cells to an M1 phenotype, visibly and quantifiably different from unstimulated cells.

### 3.2 Pro‐inflammatory macrophages result in myofibroblast, but not myoblast, cell death

**FIGURE 3:**
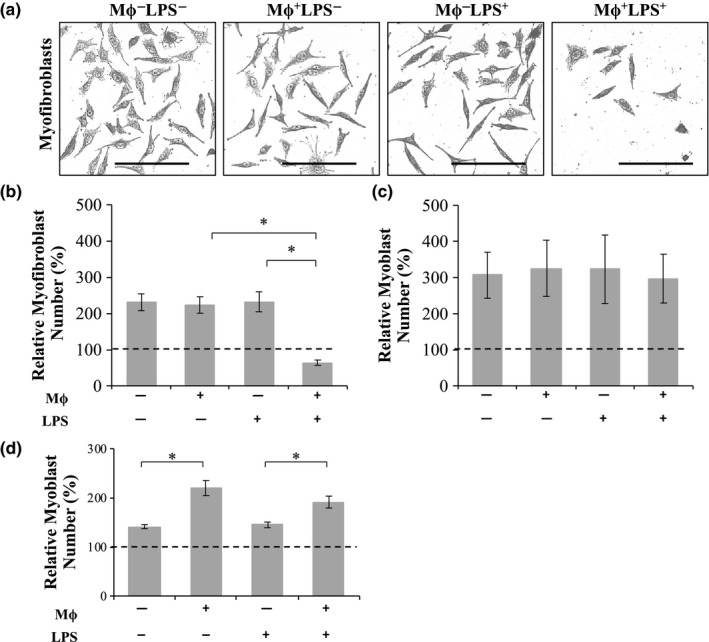
The effect of macrophages on myofibroblasts and myoblasts. Cells were cultured with or without macrophages (Mϕ; 40 × 10^3^), with or without lipopolysaccharide (LPS; 0.1 µg/ml) in 2% serum‐containing medium (b, c) or (d) SFM. (a, b) Myofibroblasts and (c, d) myoblasts were stained with Fuchsine and the relative cell numbers determined. Images were captured using an Olympus CKX41 microscope coupled to a Motic 3.0‐megapixel camera (10× objective lens; scale bar = 200 µm). *p < 0.05; *N* = 6 (myofibroblasts and myoblasts)

Macrophages were then co‐cultured with myofibroblasts or myoblasts in the presence of LPS to determine the effect of a pro‐inflammatory environment on myofibroblast and myoblast proliferation (Figure 3). Previous studies using our co‐culture technique confirmed that macrophages (and fibroblasts) do not significantly migrate from the outer edge to the center of the well within 24 h; this is relevant given the highly motile nature of macrophages (Venter & Niesler, 2018b).

Without intervention, culture conditions favored proliferation of myofibroblasts and myoblasts. Myofibroblasts alone (control condition: Mϕ^−^LPS^−^; Figure 3a,b) were present with a relative cell number (compared to the number of cells originally plated out) of 230 ± 23%. When the cells were cultured in the presence of M0 macrophages (Mϕ^+^LPS^−^) or LPS alone (Mϕ^−^LPS^+^) myofibroblast numbers were not significantly different from the control condition (232 ± 27% and 223 ± 22%, respectively). However, co‐culture with M1 macrophages (Mϕ^+^LPS^+^) caused a significant decrease (*p* < 0.05) in myofibroblast numbers to 64 ± 7% which was below the number of cells originally plated (represented by 100%), suggesting possible cell death (Figure 3b). In 2% SCM culture conditions, myoblasts alone (control condition: Mϕ^−^LPS^−^; Figure 3c) were present with a relative cell number of 306 ± 63% after 24 h and displayed no significant difference in the response over time when cultured in the presence of M0 macrophages (Mϕ^+^LPS^−;^ 325 ± 78%) or LPS (Mϕ^−^LPS^+;^ 323 ± 95%), respectively. Furthermore, myoblasts cultured in the presence of M1 macrophages (Mϕ^+^LPS^+^) were present with a relative cell number of 297 ± 68% which was also not significantly different to myoblasts cultured with LPS alone. M0 macrophages (Mϕ^+^LPS^−^) themselves (Figure 3d) continued to significantly increase (*p* < 0.05) relative myoblasts numbers from 141 ± 4% to 220 ± 15% in SFM as previously shown (Venter & Niesler, 2018b). Myoblasts maintained in the presence of LPS (Mϕ^−^LPS^+^) were present with relative cell numbers of 145 ± 6% which was not significantly different compared to the control. Co‐culture with M1 macrophages (Mϕ^+^LPS^+^) still allowed for significantly increased relative myoblast numbers to 191 ± 12%, but this was not significantly different compared to co‐culture with M0 macrophages alone (Mϕ^+^LPS^−^).

### 3.3 Mechanism of pro‐inflammatory macrophage‐mediated myofibroblast death

**FIGURE 4:**
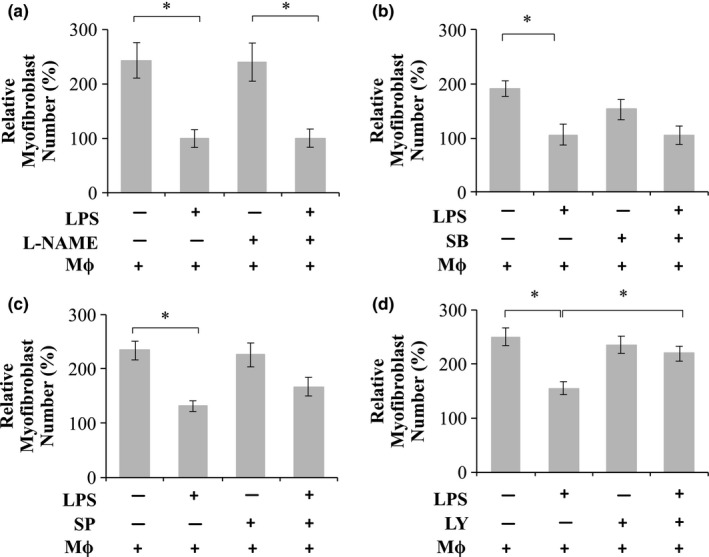
Evaluating the mechanism of pro‐inflammatory macrophage‐mediated myofibroblast death. Myofibroblasts were co‐cultured with macrophages (Mϕ; 40 × 10^3^) with or without LPS (0.1 µg/ml) with or without the inhibitors (a) L‐NAME (200 µM), (b) SB203580 (SB; 10 µM), (c) SP600125 (SP; 10 µM), (d) LY294002 (LY; 10 µM), and the relative cell numbers determined. *p < 0.05; *N* = 4–7

**FIGURE 5:**
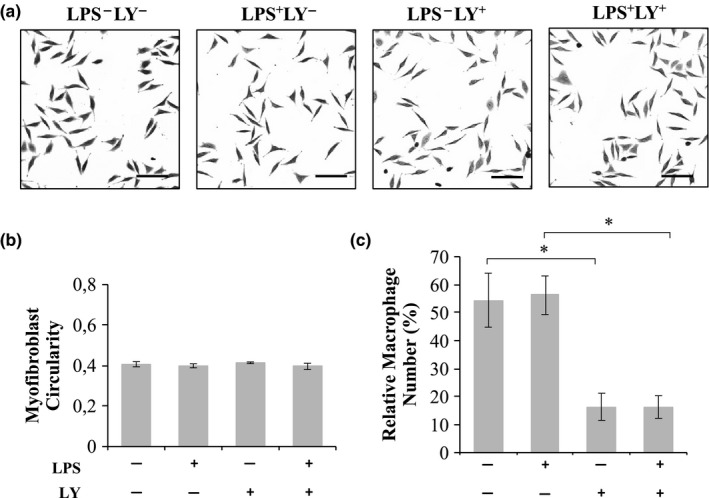
The effect of LY294002 on myofibroblast circularity and macrophage numbers. (a, b) Myofibroblasts co‐cultured with macrophages or (c) macrophages alone cultured with or without LPS (0.1 µg/ml) pre‐treatment in the presence or absence of the inhibitor LY294002 (10 µM). (a) Images were captured using an Olympus CKX41 microscope coupled to a Motic 3.0‐megapixel camera (4× objective lens; scale bar =200 µm) and (b) the average myofibroblast circularity and (c) macrophage numbers determined. *p < 0.05; *N* = 4

The mechanism by which M1 macrophages decrease myofibroblast numbers was assessed by co‐culturing macrophages and myofibroblasts in the presence of LPS and 2% SCM in the presence or absence of soluble inhibitors (Figure 4). DMSO itself showed no significant effect on macrophage or myofibroblast numbers (data not shown). Addition of L‐NAME, an iNOS inhibitor (Figure 4a), p38 MAPK inhibitor SB203580 (SB) (Figure 4b), or JNK inhibitor SP600125 (SP) (Figure 4c) showed no significant ability in rescuing myofibroblast cell loss in response to M1 macrophages. However, addition of LY294002 (LY), a PI3K inhibitor, to a co‐culture of myofibroblasts and M1 macrophages significantly (*p* < 0.05) rescued myofibroblasts from 156 ± 12% (LY^−^LPS^+^) to 219 ± 14% (LY^+^LPS^+^; Figure 4e); incubation of myofibroblasts with LY294002 (LY^+^LPS^−^) were at relative cell numbers of 236 ± 16% which was not significantly different from 250 ± 16% when the cells were cultured in the absence of LY294002 (LY^−^LPS^−^). Addition of LY294002 to a co‐culture of myofibroblasts and M1 macrophages, however, had no effect on myofibroblast circularity (Figure 5a,b). This suggested that altered myofibroblast differentiation status was not a possible mechanism by which LY294002 prevented the M1 macrophage‐mediated decrease in myofibroblast numbers.

The effect of LY294002 on macrophage numbers was also evaluated (Figure 5c). M0 and M1 macrophages had relative cell numbers of 54 ± 9.6% (LY^−^LPS^−^) and 56 ± 6.9% (LY^−^LPS^+^), respectively, which were not significantly different. However, macrophages cultured in the presence of LY294002 displayed significant decreases in cell numbers of 16 ± 4.9% (LY^+^LPS^−^) and 16 ± 4% (LY^+^LPS^+^).

## 4 DISCUSSION

Myoblasts are responsible for myogenesis after skeletal muscle injury, and their behavior is influenced by other cells residing and infiltrating the niche (Bentzinger et al., 2013). Fibroblasts are crucial at various stages of muscle regeneration (Murphy et al., 2011). Important roles for fibroblasts include their influence on proliferation, migration, and differentiation of myoblasts (Venter & Niesler, 2018a, 2018b) as well as *de novo* synthesis of the basal lamina (Sanderson et al., 1986). Similarly, macrophages and their sub‐types play their independent roles and influence myoblasts at different stages of muscle regeneration (Tidball & Villalta, 2010; Villalta et al., 2009). These non‐myoblast cells also communicate with each other providing a complex milieu of cell‐cell communication. Given this complexity *in vivo* (Cornelison, 2008), there is a role for in vitro co‐culture experiments to better identify aspects of such communication. Here we discuss the main findings that pro‐inflammatory macrophages have a greater influence on myofibroblast numbers than myoblasts in our model. We further identified quantifiable and sensitive changes in myofibroblast morphology upon exposure to the pro‐inflammatory macrophages. Finally, the PI3 kinase pathway emerged as important in the context of macrophage‐myofibroblast communication.

These cells display characteristic morphologies representative of their phenotypes. However, their phenotype may change in response to molecular stimuli. Human adipose‐derived mesenchymal stem cells stimulated to differentiate to myofibroblasts with TGF‐β acquire a more spread‐out morphology in vitro (Desai et al., 2014). Similarly, porcine dermal cells were initially spindle‐shaped when cultured and acquired polygonal shapes as they naturally differentiated to myofibroblasts over time in vitro (Khouw et al., 1999). Unstimulated murine bone marrow‐derived and human peripheral blood macrophages have been shown to acquire dendritic protrusions or elongate when stimulated to M1 or M2 macrophages in vitro (McWhorter et al., 2013; Ploeger et al., 2013). Morphology is often qualitatively represented with microscope images but rarely quantified; primary cells may yield inconsistent results when isolated from individual donors. We therefore first sought to establish different populations of fibroblasts and macrophages (using immortalized cells lines) and then characterize their morphology using cell shape and cell‐specific marker expression. We then proceeded to rapidly and quantitatively assess morphology using the image processing software ImageJ with circularity as a cell shape descriptor.

We observed that myofibroblasts maintained in 10% SCM had a short and wide morphology and were consistently positive for high levels of α‐SMA expression; these cells dedifferentiated to fibroblasts when the media was switched to 0% SCM (i.e., SFM) as evidenced by a decrease in α‐SMA expression along with attainment of a long and narrow morphology. Furthermore, we observed an intermediate morphology that also expressed low levels of α‐SMA in response to 2% SCM; this is characteristic of the proto‐myofibroblasts' phenotype within the pool of myofibroblasts (Tomasek et al., 2002). Our qualitative observations of cell shape were confirmed when we showed that myofibroblasts quantifiably had the highest circularity which decreased progressively en route to the elongated fibroblast phenotype. Therefore, a new tool has been developed that displays sensitivity to phases of cellular change of myofibroblasts in vitro. The plasticity of macrophages was further investigated in vitro by establishing populations of M0 and M1 macrophages. We observed that macrophages displayed a high circularity, but when M0 macrophages (expressing low levels of CD86) were stimulated with LPS to yield M1 macrophages (expressing high levels of CD86), the cells acquired elongated protrusions with slightly decreased circularity.

We have previously investigated the effect of myofibroblasts on myoblast fusion and showed that while high myofibroblast numbers inhibited fusion, low cell numbers were beneficial and allowed for better fusion (Venter & Niesler, 2018a). Myofibroblast numbers are typically regulated by M2 macrophages during the final stages of wound repair which promote the activation and proliferation of myofibroblasts (Garg et al., 2015; Mann et al., 2011; Ploeger et al., 2013). Since M1 macrophage‐secreted factors can result in myofibroblast death (Huang et al., 2009; Humphreys & Wilson, 1999; Nascimento et al., 2015), the prolonged presence of M1 macrophages would negatively impact myoblast differentiation and fusion and other myofibroblast functions in the late phases of regeneration, such as matrix remodeling (Mann et al., 2011). We therefore sought to develop a pro‐inflammatory co‐culture model which could be used to rapidly screen for compounds that could prevent an M1 macrophage‐mediated decrease in myofibroblast numbers. We observed that the co‐culture of M1 macrophages resulted in a significant decrease in myofibroblast numbers. This suggests that LPS interacts with macrophages to stimulate an M1 phenotype, thus satisfying the criteria for a pro‐inflammatory co‐culture environment, which in turn caused a decrease in myofibroblast numbers. This M1 macrophage‐induced decrease in numbers was specific to myofibroblasts, as the same effect was not observed with myoblasts. This is consistent with the observation that M1 macrophages increase proliferation and prevent apoptosis of myoblasts during muscle regeneration (Arnold et al., 2007; Sonnet et al., 2006).

Macrophages secrete soluble factors, such as NO, TNF‐α, and PGE_2_, which result in myofibroblast death (Huang et al., 2009; Humphreys & Wilson, 1999; Nascimento et al., 2015). Compounds which could inhibit this effect may intervene by different methods such as inhibiting pathways within macrophages which reduced secretion of these factors or by inhibiting pathways within myofibroblasts to promote survival. Since it is unclear in which cells these compounds may be exhibiting their effects, it becomes beneficial to screen for compounds in the presence of both cell types, for example when M1 macrophages are in co‐culture with myofibroblasts. We therefore used our previously developed co‐culture method to screen for inhibitors which could prevent M1 macrophage‐mediated decrease in myofibroblasts numbers. We found that L‐NAME (an iNOS inhibitor), SB203580 (a p38 MAPK inhibitor), and SP600125 (a JNK inhibitor) were unable to prevent the loss in myofibroblast numbers. Only the PI3K inhibitor LY294002, however, was able to prevent a loss in myofibroblast numbers. In addition, the PI3K inhibitor decreased macrophage cell number; this suggests that blocking of this pathway is sufficient to rescue myofibroblast numbers. The effect is therefore not direct, but via a decrease in M0 and/or M1 macrophage numbers. The PI3K/Akt pathway is an important cell survival pathway in macrophages and inhibition via LY294002 has previously resulted in macrophage death (Liu et al., 2001).

## 5 CONCLUSION

We have established different phenotypes of fibroblasts and macrophages in vitro, characterized them with phenotype‐specific markers, and successfully used ImageJ to measure circularity as a quantitative assessment of morphology. These cells displayed changes in circularity associated with their phenotypes, as previously observed, because of the experimental conditions. Unstimulated macrophages showed a decrease in circularity when activated to M1 macrophages in response to LPS. Myofibroblasts also displayed a decrease in circularity when cultured in media without serum and de‐differentiated to fibroblasts. Therefore, circularity can be used as a tool to rapidly assess changes in cell morphology; in this study, we offer a simple quantitative means to assess the changes in morphology in vitro.

We also adapted our previously developed co‐culture model to represent a pro‐inflammatory model to assess the cross‐talk between cells present in the satellite cell niche during muscle regeneration so that we could rapidly screen for inhibitors which could possibly prevent M1 macrophage‐mediated decrease in myofibroblast numbers. The addition of PI3K inhibitor could rescue myofibroblast cell numbers; potentially by causing a decrease in macrophage numbers. Although, we showed that the inhibitors we used had no effect on morphology, circularity could still be used as a tool to evaluate the effect of experimental conditions on cells (such as fibroblasts in SCM). As a next step toward physiological relevance, the inhibitor of interest could be evaluated using primary cells. In conclusion, our current pro‐inflammatory co‐culture model is a useful tool to rapidly screen for a range of therapeutic agents which can prevent M1 macrophage‐induced decreases in myofibroblasts.
